# The Limits of Cognitive Reappraisal: Changing Pain Valence, but not Persistence, during a Resistance Exercise Task

**DOI:** 10.3390/ijerph16193739

**Published:** 2019-10-04

**Authors:** Catherine J. Berman, Julia D. O’Brien, Zachary Zenko, Dan Ariely

**Affiliations:** 1 Center for Advanced Hindsight, Duke University, Durham, NC 27701, USA; julie.obrien@duke.edu (J.D.O.); dan@danariely.com (D.A.); 2 Department of Kinesiology, California State University, Bakersfield, CA 93311, USA; zzenko@csub.edu

**Keywords:** reappraisal, exercise, affect, valence, arousal, exertion, pain, discomfort, persistence, resistance

## Abstract

Physiological discomfort is commonly cited as a barrier for initiating and persisting with exercise. Although individuals may think of physiological discomfort as determined by physical sensations, it can also be influenced by cognitive and emotional factors. We explored the impacts of interpreting the purpose of pain as a sign of muscle building (helpful) vs. a sign of muscle tearing and possible injury (harmful) and tested the effect of cognitive reappraisals, or shifting interpretations of pain, on exercise persistence and the subjective experience of discomfort during exercise. Seventy-eight participants were randomized to listen to voice recordings that framed exercise-related pain as helpful vs. harmful before participating in a standard muscular endurance test using the YMCA protocol. Although the two experimental groups did not differ in the overall number of resistance training repetitions achieved, participants who were asked to think about the benefits (rather than the negative consequences) of pain reported less negative pain valence during exercise. Thus, the experience of pain was influenced by appraisals of the meaning of pain, but differences in pain valence did not impact exercise persistence. Theoretical implications and applications for affect-based exercise interventions are discussed.

## 1. Introduction

Despite many well-known benefits of regular physical activity, most adults in the USA [1,2] and Canada [3,4] fail to meet physical activity guidelines, although estimates vary depending on the inclusion of lifestyle activities [5]. Across high-income countries, rates of physical inactivity are rising [6].

Insufficient physical activity leads to severe public health problems, increasing the risk of major non-communicable diseases and associated financial burdens [7]. Physical inactivity is associated with musculoskeletal conditions (e.g., osteoporosis, lower back pain, rheumatoid arthritis), cardiorespiratory conditions, diabetes, cancer (colon and breast cancer), and neurological and cognitive disorders [8]. In China, for instance, physical inactivity is estimated to be responsible for over 15% of the medical and non-medical costs of five major non-communicable diseases in the country: coronary artery disease, stroke, hypertension, cancer, and type 2 diabetes [9]. In addition to global health and financial consequences of physical inactivity, 13.4 million Disability Adjusted Life Years (DALYs) were attributed to physical inactivity in 2013 [10]. 

Although most people recognize the benefits of exercise, many (especially those who are currently inactive) find that physical activity is unpleasant or painful [11]. Indeed, discomfort associated with exercise is reported as a barrier to exercise adherence [12,13]. Rather than being a fully deliberative, rational process that is driven by knowledge of benefits of an active lifestyle or consequences of a sedentary lifestyle, exercise is a behavior that is highly influenced by pleasure and displeasure experienced during exercise [13,14]. Anticipated affect and enjoyment are associated with initiating physical activity for physically inactive adults, as well as exercise adherence for adults who are already active [15]. Further, pleasure experienced during exercise predicts self-reported future exercise behavior at 6-month (*r* = 0.50) and 12-month (*r* = 0.47) follow-ups [13,16,17]. 

Perceptions of pain and physical discomfort are highly subjective and impacted by emotions and beliefs [18]. In the domain of exercise, affective responses arise from an interaction between interoceptive cues (e.g., muscular or respiratory sensations) and cognitive factors (e.g., self-efficacy, the meanings ascribed to sensory information, and attention) according to dual-mode theory [14,18,19,20]. Further, the intensity of exercise determines the relative impact of interoceptive versus cognitive cues on affective experiences: cognitive factors play a greater role at lower exercise intensities, whereas interoceptive cues dominate at higher intensities [19,20,21,22]. 

Because unpleasant affective experiences are common, particularly during higher-intensity exercise above the ventilatory or lactate threshold [23], changing the affective experience of exercise is an important area for intervention. It has been demonstrated that intensity and pleasure are negatively correlated [21], and that as intensity increases, people gradually shift to paying more attention to the sensory experience of exercise rather than external environmental stimuli [24,25]. Numerous studies have shown that this associative focus is related to decreased pleasure [26].

In the broader psychological literature, emotion regulation describes the processes used to change emotional experiences (including the type, duration, and magnitude of emotions), as well as the physiological, cognitive, and behavioral responses to emotions [27]. Previous work on emotion regulation strategies for exercise has largely focused on dissociative focus through distraction via music and virtual reality videos, although these interventions have shown somewhat mixed results. Some of these studies have demonstrated that music or video during exercise is associated with psychological and physiological benefits: reduced perceived exertion (during submaximal exercise), improved overall affective experiences, and increased exercise intensity (particularly when synchronized music or self-selected music are used) [28,29,30,31,32,33]. In contrast, other studies have shown no impact of music and videos on affective measures, despite positive effects on exercise intensity and enhanced adherence [34,35]. 

One proposed mechanism for the positive effects of music and virtual reality is distraction or attentional dissociation, defined as an external attentional focus directed toward distracting environmental cues and away from internal sensory cues of fatigue and discomfort [28,36,37]. In the broader physical activity literature, these attention strategies are classified as “dissociative” (distancing oneself from physical sensations), whereas “associative” emotion regulation strategies involve paying attention to physical sensations [38].

In addition to distraction techniques, associative interventions may also be an effective emotion regulation strategy to increase exercise pleasantness. One widely studied associative technique is cognitive reappraisal, or changing how one interprets the meaning of an emotion before it is fully experienced in order to alter its emotional impact [27,39,40,41,42,43]. The benefits of reappraisal have been demonstrated in many domains; for example, reappraisal is associated with lower rates of depression, increased self-reported life satisfaction, and lower levels of negative affect [44,45,46]. Reappraisal has also been shown to have physiological benefits, including reducing physiological arousal, increasing cardiovascular efficiency, and decreasing vascular resistance during psychologically stressful tasks [47,48,49]. 

In the domain of exercise, reappraisal is largely unstudied as an intervention to attenuate pain, change perceptions of exercise discomfort, and enhance exercise behavior. We are aware of only one study that has experimentally tested cognitive reappraisal during exercise; researchers found that a cognitive reappraisal manipulation reduced arousal and perceived exertion, but not feelings of pleasure or displeasure. The cognitive reappraisal manipulation also failed to influence heart rate and distance run during the endurance-exercise task [50]. 

Given the lack of experimental research examining the impacts of reappraisal on resistance exercise behavior, we aimed to test how different interpretations of pain (as helpful or harmful) during exercise impact persistence during a challenging muscular endurance task and desire to repeat the task. We hypothesized that compared to participants in the “Harmful” group, participants in the “Helpful” group would have greater exercise persistence (H1) and more frequently report a desire to repeat the exercise task (H2). Further, we expected that participants in the “Helpful” group would experience greater affective valence, less negative pain valence, lower pain intensity, lower exercise-task anxiety, and lower arousal (H3).

## 2. Materials and Methods 

### 2.1. Participants

After obtaining ethical approval, participants ages 18-55 were recruited through an online recruitment platform (SONA system). Prior to any laboratory visits, interested participants were sent a consent form, as well as the Physical Activity Readiness Questionnaire (PAR-Q) [51] to assess health risks for physical activity tasks. If participants answered any of these health risk questions affirmatively, they were disqualified from the study as a safety precaution. Participants provided their informed consent before participating in the study, and the Institutional Review Board (IRB) at Duke University approved all study activities.

A power analysis using *desire to repeat the exercise task* as a dependent variable indicated that 88 participants were needed to achieve 80% statistical power with a Type 1 error rate of 5%, anticipating a medium effect size (*w* = 0.3). The target sample was inflated by 12% to account for potential dropout. Although 96 participants expressed interest, 18 did not complete the study because they did not schedule or attend their laboratory visit. Therefore, 78 participants completed the study and outcome measures (55 women, 23 men; mean age: 28.25 [SD: 9.95] years). The sample of 78 participants was deemed to yield sufficient power (>99%) to detect a medium effect size (*f* = 0.25), with a Type 1 error rate of 5% in a 2 (group) × 2 (time) RMANOVA, with correlated repeated measures (*r* = 0.7). Study data are publicly available at https://osf.io/tpe6z/; per journal guidelines, demographics that are not relevant to the study hypotheses have been removed to preserve participants’ anonymity.

### 2.2. Measures

Persistence during the exercise task was measured by counting the number of repetitions during the bench-press task (described below). This task served as a behavioral measure to determine if the experimental manipulation had an impact on an objective measure of physical activity.

To measure affective valence, the valenced pleasure-displeasure dimension of Russell’s circumplex model of affect (1980) [52] was measured using the Feeling Scale (FS) [53]. The FS is a single-item, 11-point, bipolar rating scale used to measure affective valence. The scale ranges from “very bad” (−5) to “very good” (+5), with “neutral” (0) as a midpoint and contains verbal anchors at odd numbers. Hardy and Rejeski (1989) have reported concurrent validity data [53]. 

Arousal was measured using the Felt Arousal Scale (FAS) [54], a single-item, six-point rating scale from “low arousal” (1) to “high arousal” (6) to measure perceived activation, one of the dimensions of Russell’s (1980) circumplex model of affect [52]. 

Pain intensity was measured using a rating scale originally developed to measure pain during cycle ergometry [55]. The scale contains 0-10 numbers with the following verbal anchors: 0 = “no pain at all,” ½ = “very faint pain (just noticeable),” 1 = “weak pain,” 2 = “mild pain,” 3 = “moderate pain,” 4 = “somewhat strong pain,” 5 = “strong pain,” 7 = “very strong pain,” and 10 = “extremely intense pain (almost unbearable).” The scale ends with an unnumbered category (“unbearable pain”) to serve as a maximal anchor.

Pain valence was measured using a bipolar scale from “most unpleasant imaginable” to “most pleasant imaginable,” with “neutral” serving as a midpoint. The scale contained the following evenly spaced verbal anchors on each side of the scale: extremely, strongly, moderately, mildly, slightly, and barely. Participants’ responses were coded from −100 to +100 based on ruler measurements. This scale was only presented if the participant reported some pain, as indicated by the pain intensity scale; thus, these data were not collected for nine participants. This scale was based on the Empirical Valence Scale [56]. Although we expected the affective experience of pain to be inherently unpleasant, this measure was designed to assess the cognitively mediated emotional memory of pain (e.g., “it hurt but felt good”). Therefore, the scale was bipolar and included the possibility of positive responses (although only 12.5% rated the pain as more pleasant than neutral).

Anxiety was measured using a five-item rating scale from “not at all” (1) to “very much” (5) with the following items: 1. How nervous are you about the exercise? 2. How much risk are you taking by exercising right now? 3. Do you believe that the exercise might harm you? 4. How threatened do you feel by the exercise? 5. How anxious do you feel as you prepare to exercise? These items were designed to assess the level of cognitively appraised threat associated with exercise, which is an essential defining feature of state anxiety [57]. Internal consistency of this measure was acceptable in the current sample (Cronbach’s α = 0.78). 

Participants completed a demographics questionnaire. These questions included self-reported age, gender, education, race and ethnicity, household income, marital status, number of children, and weekly exercise behavior based on the International Physical Activity Questionnaire Short Form [58] (see Table 1 and Table 2 for demographics by group). 

Finally, participants’ *desire to repeat the exercise* task was measured. After the participant completed all surveys, the researcher asked the participant, “Would you like to do the task again?” and recorded the response as either yes or no.

### 2.3. Procedure

#### 2.3.1. Study Introduction

The study procedures were conducted in a university behavioral laboratory, a minimally decorated office setting with the requisite exercise equipment for the study. During a laboratory visit, eligible participants signed an informed consent document. When explaining the purpose of the study, a research assistant used a cover story in order to mitigate potential demand effects. Specifically, participants were told that they would be asked to provide feedback on an audio recording for an exercise app, per the following script:

*“We are developing a smartphone app that will guide users through exercise sessions. We are interested in your feedback about the app to help us develop the content. This app includes written text, visuals, and an audio component. We’d like you to review the audio component of the app. First we’ll do a warm-up exercise, and then we’ll have you listen to the app right before you exercise. Then we’ll ask you to evaluate your experience.”*

#### 2.3.2. Warm-up Exercise Task

Prior to the first exercise task, researchers asked participants to bench press a bar only (with no weights) six times and then rest for one minute. This step was included to ensure that participants were familiar with the motion required for a bench press. The participant rested for one minute following the task.

#### 2.3.3. Baseline Exercise Task

The YMCA protocol, a standard procedure used to test muscular endurance, was used for exercise tasks. The researcher asked the participant to bench press weights as many times as possible to the beat of a metronome of 60 beats per minute, or 30 lifts per minute (1 beat up, 1 beat down). The researcher demonstrated how to safely use the bench press, by lifting at the pace of the metronome, extending upwards with arms fully extended, and bringing the bar to the chest. The test ended when participants could no longer keep the pace of the metronome. The participant rested for three minutes following this exercise. The participant rested for three minutes after this task.

Men completed the task with 80 lb (36 kg) weights, and women completed the task with 35 lb (16 kg) weights. For any participant who could not complete one repetition, the weight was dropped by 5 to 10 pounds at a time until the participant could complete a repetition. 

#### 2.3.4. Intervention

While the participant was resting, the researcher asked the participant to listen to a 31-second recorded introduction to the “app” explaining how exercise impacts the body. The participant was randomized to listen to the “Helpful” or “Harmful” recording. This research was conducted over several weeks; each participant was fully randomized to condition to eliminate systematic effects of day or time across conditions.

#### 2.3.5. “Helpful” Condition

Participants in the “Helpful” group heard an audio recording with the following script that framed exercise-related pain as potentially helpful to the body:

*“We want you to be informed about how your body responds to resistance exercise. Every time you push yourself and feel some pain or discomfort, your muscle fibers are stretching slightly, causing strengthening in the proteins in your muscles. Your body is experiencing muscle expansion and growth. The pain and discomfort you feel indicates that you are pushing yourself sufficiently and may help your physical functioning and quality of life.”*

#### 2.3.6. “Harmful” Condition

Participants in the “Harmful” group heard a similar audio recording that instead framed exercise-related pain as potentially harmful to the body:

*“We want you to be informed about how your body responds to resistance exercise. Every time you push yourself and feel some pain or discomfort, your muscle fibers are tearing slightly, causing trauma in the proteins in your muscles. Your body is experiencing a stress response and inflammation. The pain and discomfort you feel indicates that you are pushing yourself a lot and may harm your physical functioning and quality of life.”*

#### 2.3.7. Follow-up Exercise Task

Immediately after listening to the recording, the researcher asked the participant to repeat the previous exercise task and bench press the weights as many times as possible to the beat of the metronome (using the same YMCA protocol). The number of repetitions performed, controlling for baseline repetitions, functioned as a measure of persistence. The participant then rested for three minutes.

#### 2.3.8. Dependent Measures

After completing the exercise task, participants were asked to complete surveys measuring affective valence, arousal, pain intensity, pain valence, exercise-task anxiety, and demographics; the researcher ended by asking the participant if they wanted to do the exercise task again and recorded this response. Regardless of the participant’s answer, the study was completed and participants were thanked and compensated for their time.

## 3. Results

### 3.1. Behavioral Outcomes

First, to test the effect of our experimental manipulation on the number of repetitions performed in the second round of the task, we conducted a 2 (group: Helpful reframing vs. Harmful reframing) × 2 (time: baseline vs. follow-up) RMANOVA to determine if the Helpful reframing resulted in a greater number of repetitions performed during the follow-up exercise task. There was no significant effect of group, *F*(1, 79) = 0.032, *p* = 0.858, η_p_^2^ < 0.001. There was a significant effect of time *F*(1, 79) = 13.074, *p* = 0.001, η_p_^2^ = 0.142. Most importantly for the hypothesis that the Helpful reframing would lead to greater exercise persistence, there was no significant or meaningful group by time interaction, *F*(1, 79) = 0.091, *p* = 0.763, η_p_^2^ = 0.001. Thus, H1 was not supported. Similarly, the Chi-Squared analysis revealed no significant difference between groups in the frequency to express a desire to repeat the exercise task (χ^2^ = 0.56, *p* = 0.454). Specifically, 20 of 38 participants in the Harmful group expressed a desire to repeat the task, whereas 25 out of 41 participants in the Helpful group expressed a desire to repeat the task. Thus, H2 was not supported.

### 3.2. Psychological Outcomes

Finally, a MANOVA to determine if there were significant group differences on the outcomes of affective valence, pain valence, pain intensity, exercise-task anxiety, and arousal was performed. The multivariate test was significant (V = 0.162, *F*(5, 64) = 2.48, *p* = 0.041, η_p_^2^ = 0.162). Univariate tests revealed no significant effect of framing manipulation on affective valence, *F*(1, 68) = 0.118, *p* = 0.732, η_p_^2^ = 0.002; no significant effect on pain intensity, *F*(1, 68) = 1.125, *p* = 0.293, η_p_^2^ = 0.016; no significant effect on exercise-task anxiety, *F*(1, 68) = 1.312, *p* = 0.256, η_p_^2^ = 0.019; and no significant effect on arousal, *F*(1, 68) = 1.226, *p* = 0.272, η_p_^2^ = 0.018. There was, however, a significant effect on pain valence, such that the Helpful reframing group reported less unpleasantness associated with pain, *F*(1, 68) = 5.103, *p* = 0.027, η_p_^2^ = 0.070. Thus, H3 was partially supported. Values for each of these dependent variables are displayed in Table 3.

## 4. Discussion

This study provided the first direct test of cognitive reappraisal on exercise persistence. Seventy-eight participants completed a resistance exercise task and were then randomized to listen to voice recordings designed to frame exercise discomfort as helpful (i.e., a sign of muscle building) or harmful (i.e., a sign of muscle tearing and possible injury). Participants then repeated the task to determine if these divergent mental models of pain would result in changes in exercise persistence. We hypothesized that compared to participants in the Harmful group, participants in the Helpful group would show greater exercise persistence and more frequently report a desire to repeat the exercise task; we also expected participants in the Helpful group to show greater affective valence, less negative pain valence, lower pain intensity, lower exercise-task anxiety, and lower arousal. 

Our hypotheses were partially supported. There was no evidence that the Helpful condition led to greater persistence or a greater desire to repeat the exercise task, compared to the Harmful condition. There was also no effect of condition on affective valence, pain intensity, exercise-task intensity, or arousal. However, there was a significant effect of framing on pain valence, whereby participants in the Helpful condition reported less negative pain valence than those in the Harmful condition. Thus, positive cognitive reappraisal, which framed exercise pain as helpful, led participants to rate the pain they experienced as less unpleasant than negative cognitive reappraisal, which framed exercise pain as harmful. 

The improvement in pain valence suggests it is possible to change the cognitive label that people ascribe to exercise-related pain even without changing the intensity or physiological nature of the exercise session. Participants in both conditions were able to persist with the exercise session, but those in the Helpful condition felt less negatively about the pain they experienced. This finding adds to growing literature suggesting that affective experiences of exercise can be impacted through psychological interventions without changing the physiological aspects of the exercise [59]. Furthermore, this experiment illustrates that a minimally intrusive intervention (listening to a brief reappraisal audio recording for approximately 30 seconds) can have positive impacts on pain valence in exercise. 

Previous research suggests that improved pain valence may have downstream benefits. For instance, increased use of pain reappraisal techniques is correlated with increased use of other emotion regulation techniques such as mindfulness [60,61]. Thus, cognitive reappraisal may be used in concert with other emotion regulation techniques that together create an ‘upward spiral,’ consistent with Fredrickson’s broaden-and-build theory of positive emotions [61,62]. Furthermore, pain reappraisal could lead to lower levels of pain catastrophizing, or the tendency to exaggerate the threat of pain and the inability to inhibit pain-related thoughts [63]. Cognitive-behavioral models of pain, such as the fear avoidance model [64], suggest that pain catastrophizing could cyclically increase attention to pain, somatic awareness, perceived threat, fear, and behavioral avoidance [65]. Thus, cognitive reappraisal could interrupt the cycle of catastrophizing by providing more adaptive interpretations of pain that do not increase perceptions of threat.

Reduced pain catastrophizing has been shown to impact exercise behavior: for example, among those with high anxiety sensitivity, pain catastrophizing has been shown to mediate the relation between self-reported strenuous exercise bouts and pain [66]. Catastrophizing has also been shown to predict reductions in weight lifted in response to delayed onset muscle soreness (DOMS) [67]. Thus, it is possible that cognitive reappraisal interventions that improve pain valence could positively impact responses to DOMS and long-term exercise behavior by decreasing pain catastrophizing. Future longitudinal research can examine the long-term impacts of reappraisal outside of an acute exercise session. Specifically, longitudinal studies can test potential psychological and behavioral benefits of training individuals to distinguish between pain that indicates a productive workout and subsequent muscle growth versus pain that indicates possible injury.

Although cognitive reappraisal manipulations led to improved pain valence, these affective benefits did not impact overall affective valence or exercise persistence. Prior research offers potential explanations for these null effects. First, as task challenge increases to near-maximal efforts, cognitive reappraisal is theorized to have limited effects [20,29]. At higher exercise intensities, physiological cues play a greater role than cognitive cues in impacting affective valence [21,68]. Neurological evidence also supports this finding: at low to moderate intensities of exercise, there is increased activity in the prefrontal cortex (PFC) [69,70,71,72,73,74], which is responsible for neural processes related to cognitive reappraisal, perceived control over pain, or other emotion regulation strategies and cognitive abilities. In contrast, at higher exercise intensities (in resistance training and aerobic exercise alike), there is lower neural activity in the PFC [39,75,76,77,78]. Thus, because physically demanding tasks inhibit neural activity in the area of the brain responsible for implementing cognitive reappraisal processes, it is possible that too few cognitive resources are available during high intensity exercise to successfully implement cognitive reappraisal strategies and impact overall affective valence and exercise persistence. Thus, it is possible that persistence would have been affected by cognitive appraisal during a lower effort resistance-exercise task, with lower physiological strain and lower susceptibility to ceiling effects.

It is possible that distraction [29,79] may be more effective than reappraisal in the context of resistance exercise, although few studies have directly compared cognitive reappraisal to distraction in this domain. Indeed, we are aware of only one previous study [50] that has directly compared the effects of reappraisal versus distraction on affective valence. Runners in the study were randomized to three conditions while running at their 75–85% age-adjusted maximum heart rate (HRmax): prompts to cognitively reappraise discomfort (by distancing oneself from the discomfort), prompts to use distraction techniques, or no emotion regulation instruction at all (control). The reappraisal condition led to lower arousal and perceived exertion compared to control, but responses to reappraisal did not differ from responses to distraction. 

Methodological issues in this previous study make its findings difficult to compare to those of the present study. The reappraisal instructions in the previous study [50] encouraged participants to think about their exercise experiences in a neutral, detached way but did not provide new meanings that participants could apply to their discomfort; therefore, the manipulation might be more akin to emotion suppression techniques rather than cognitive reappraisal and diverges conceptually from the reappraisal manipulations used in the present study (which served to change interpretations of the consequences of discomfort). Moreover, manipulation checks failed to show differences in participants’ self-reported use of cognitive reappraisal strategies across experimental conditions, suggesting that the reappraisal manipulation was not successfully employed. Further, the participants completed an endurance exercise task, whereas the participants in the present study completed a resistance exercise task. Finally, the previous study used an active sample of runners who ran at least 30 miles per week; thus, the findings are difficult to generalize to research with inactive participants, who are most likely to benefit from this type of intervention. In summary, findings about the role of reappraisal in this earlier reappraisal study are difficult to compare to those of the present study, given methodological discrepancies between the two studies.

Methodological limitations in our own study might also explain the null effects of reappraisal manipulations on exercise persistence and interest in repeating the exercise task. First, although our study was sufficiently powered to detect medium-sized effects of manipulations, it is possible that our sample size was too small to detect smaller effects of cognitive reappraisal. Second, ceiling effects may be present, such that reappraisal is unlikely to influence task persistence on an already challenging exercise task, where baseline measurement assessed maximal effort.

Third, it is possible that the experimental context primed participants to perform a similar number of repetitions during both exercise tasks. Deception about the study’s premise was used to reduce demand effects (the experimenter stated that the primary aim of the study was to give feedback on an exercise app), so it is possible that this experimental context led participants to view the exercise session as a simulation rather than as an opportunity to fully exert themselves. Relatedly, participants might have psychologically anchored themselves on the number of repetitions they performed in the baseline tasks due to consistency bias, which may have motivated participants to perform a similar number of repetitions during the post-manipulation exercise task. This mindset may have been reinforced by language used in the Helpful condition, which stated that sensations of pain indicated that participants were exerting themselves “sufficiently,” and these instructions may have primed participants to not exceed baseline levels of exertion. Furthermore, because the invitation to repeat the exercise task was framed as voluntary, it is possible that most participants declined to repeat the task because it was perceived as a non-essential part of the study protocol.

Lastly, it is possible that differences in participants’ weekly exercise activity impacted the results, since the sample included both regularly and insufficiently active individuals, based on self-reported behavior. Previous research has shown that exercise contributes to enhanced cognitive functioning and performance on the Cognitive Reappraisal Task, which assesses the ability to reappraise general negative emotions [80]. Thus, it is possible that participants with higher levels of weekly exercise had greater cognitive functioning and ability to reappraise pain, which could have eclipsed the effects of the experimental manipulations. Additionally, differences in participants’ previous experience with resistance training may have impacted findings. Although participants reported their average amount of weekly exercise, participants were not asked about their familiarity with a resistance training activity or about their typical mode of exercise. Because resistance training can involve precise and technical movements, it is possible that participants with less experience with the task may have had more difficulty with it, and that differences in task familiarity may have impacted the results.

In summary, improvements in pain valence may have important implications for reducing pain catastrophizing. This study supports existing theories about the relation between physiological demand and cognitive processes, suggesting that at high levels of exertion in an acute exercise session, high physiological arousal may impede cognitive interventions from observably impacting affective responses and exercise behavior. Finally, future research can address this study’s limitations by replicating cognitive reappraisal tasks in a DOMS context, which may be less vulnerable to consistency bias effects (given more time between exercise sessions) and may be more likely to be effective given reduced physiological arousal in DOMS compared to an acute exercise session.

## 5. Conclusions

The American College of Sports Medicine recognizes that pain can be a significant barrier to initiating and maintaining an exercise routine [81], and addressing pain in exercise is an understudied area for intervention. As Ekkekakis (2017) highlighted, exercise psychology has not devised methods for increasing physical activity at a large scale, and so “researchers are called to invest time and effort into investigations designed to develop and test methods of making the experience of exercise and physical activity more pleasant” [82]. Scalable affective interventions aimed at improving exercise behavior, particularly for insufficiently active participants, are therefore an important area of research. This experiment contributes the first known study of cognitive reappraisal on resistance exercise persistence and demonstrates that positive reappraisal can lead to improved pain valence compared to negative reappraisal. Although positive reappraisal failed to improve exercise persistence or interest in repeating an exercise task (compared to negative reappraisal), theoretical explanations regarding the neural processes involved in reappraisal and high intensity exercise may explain these effects. 

Future research should examine the role of cognitive reappraisal on exercise behavior at low to moderate exercise intensities. It is possible that under less physiologically demanding tasks, reappraisal may provide beneficial effects on exercise persistence for insufficiently active people. Future research should also test the role of cognitive reappraisal on exercise persistence in more naturalistic contexts, where participants’ beliefs about the goals of an exercise session are more akin to naturalistic beliefs (e.g., to enjoy oneself, to experience health benefits, etc.). It is possible that in contexts in which participants are more likely to exercise for their own benefit (rather than under the premise of testing an app), participants would be more likely to increase exercise persistence. Given the low cost and time required for implementing this intervention, it can also be easily applied and tested in real-world settings to improve affective experiences during exercise, such as in fitness coaching programs or in digital or mobile interventions. Finally, future research can examine the role of cognitive reappraisal on long-term exercise behavior. In summary, this study tests a novel emotion regulation strategy in the domain of exercise and future research should elucidate the boundary conditions for the affective impacts of cognitive reappraisal in physical activity.

## Figures and Tables

**Table 1:** Demographics by experimental group.

Demographic	Helpful Condition	Harmful Condition
Men	14	10
Women	27	28
Grade School	1	0
High school	3	2
Some college	8	14
Undergraduate degree	18	13
Graduate degree	11	6
Post-graduate degree	0	3
Black, African American	6	8
White	18	17
Asian	12	8
Latino/a/x	4	3
Native American	1	0
Other or Missing	0	2
Single	28	27
Live with partner	2	5
Married	7	4
Divorced	2	0
Other	2	2
<$15k	6	4
$15–35k	8	12
$36–50k	8	5
$51–75k	4	4
$76–100k	5	5
$101–150k	6	4
$151–200k	1	1
$201–500k	2	2
>$500k	1	1
Mean age	28.51 (10.25) ^1^	27.97 (9.75)
Mean # of children	1.17 (0.50)	1.18 (0.73)

^1^ Parentheses indicate standard deviation.

**Table 2:** Weekly exercise demographics by experimental group (minutes per week).

Measure	Helpful Condition		Harmful Condition	
	Mean	Median	Mean	Median
Vigorous intensity exercise	134.51 (135.00) ^1^	100.00 [80.00] ^2^	116.18 (94.90)	110.00 [170.00]
Moderate intensity exercise	85.00 (65.56)	70.00 [20.00]	85.79 (82.59)	70.00 [90.00]

^1^ Parentheses indicate standard deviation. ^2^ Brackets indicate interquartile range.

**Table 3:** Means and standard deviations for affective valence, pain intensity, pain valence, exercise-task anxiety, arousal, and change in number of repetitions.

Variable	Helpful Condition		Harmful Condition		*p*-Value
	M	SD	M	SD	
Affective valence	2.14	2.09	1.97	2.01	0.732
Pain intensity	2.43	1.33	2.10	1.25	0.293
Pain valence	−12.06	3.83	−24.31	4.14	0.027
Exercise-task anxiety	7.94	2.93	8.85	3.69	0.256
Arousal	3.75	0.97	4.03	1.11	0.272
Repetitions ^1^	−3.55	6.36	−3.00	9.71	

Note: Affective valence ranges from −5 (very bad) to +5 (very good). Pain intensity ranges from 0 (“no pain”) to 10 (“extremely intense pain [almost unbearable]”). Pain valence ranges from −100 (“most unpleasant imaginable”) to +100 (“most pleasant imaginable”). Exercise-task anxiety ranges from 1 (low) to 5 (high). Arousal ranges from 1 (“low arousal”) to 6 (“high arousal”). ^1^ Difference in repetitions from baseline.
